# Characterization of the complete chloroplast genome of *Dracocephalum heterophyllum* (Lamiaceae)

**DOI:** 10.1080/23802359.2021.1945507

**Published:** 2021-07-02

**Authors:** Lei Zhang, Zhiheng Wang, Yuqing Wei

**Affiliations:** Key Laboratory of Ecological Protection of Agro-pastoral Ecotones in the Yellow River Basin, National Ethnic Affairs Commission of the People’s Republic of China, College of Biological Science & Engineering, North Minzu University, Yinchuan, Ningxia, P. R. China

**Keywords:** Lamiaceae, *Dracocephalum heterophyllum*, chloroplast genome, phylogenetic analysis

## Abstract

*Dracocephalum heterophyllum* is a medicinal plant. The complete chloroplast genome sequence is 150,860 bp in length, which contains 133 complete genes, among them, 88 are protein-coding genes (88 PCGs), 8 are ribosomal RNA genes (8 rRNAs), and 37 are tRNA genes (37 tRNAs). The overall GC content of chloroplast DNA is 37.6%, the respective values of the LSC, SSC, and IR regions are 36.0%, 31.6%, and 43.0%. Phylogenetic tree shows that *D. heterophyllum* is a sister to *D. moldavica* and *D. palmatum.*

*Dracocephalum heterophyllum* Benth. belongs to the family Lamiaceae, which is a small perennial aromatic herb and has been of medicinal importance in traditional Chinese medicine (Rakhmonovich et al. 2015). Its habitat pertains to open and moist slopes of China. In Xinjiang and Tibet, it is used as traditional medicine in treating tracheitis and cardiovascular disease (Numonov et al. 2013a, 2013b). The decoction of dried flowers and leaves is widely used to treat cold, cough, and headache (Ballabh and Chaurasia 2007). The extract of the plants can be used in cosmetics, food, and pharmaceutical industries for its antimicrobial and antioxidant properties (Zhang et al. 2008). However, the chloroplast genome of *D. heterophyllum* has not been reported. In this study, we assembled the complete chloroplast genome of *D. heterophyllum*, hoping to lay a foundation for further research.

Fresh leaves of *D. heterophyllum* were collected from Qilian (Haibei, Qinghai, China; coordinates: 98°16′E, 38°57′N) and dried with silica gel. The voucher specimen was stored in Sichuan University Herbarium with the accession number being QTPLJQCHNP0263022. Total genomic DNA was extracted with a modified CTAB method (Doyle and Doyle 1987) and a 350-bp library was constructed. This library was sequenced on the Illumina NovaSeq 6000 system with 150 bp paired-end read length. We obtained 10 million high quality pair-end reads for *D. heterophyllum*, and after removing the adapters, the remaining reads were used to assemble the complete chloroplast genome by GetOrganelle pipeline v1.6.3a (Jin et al. 2020). The complete chloroplasts genome sequence of *D. tanguticum* was used as a reference. Plann v1.1 (Huang and Cronk 2015) and Geneious v11.0.3 (Kearse et al. 2012) were used to annotate the chloroplasts genome and correct the annotations.

The total chloroplast genome length of *D. heterophyllum* (MW970109) is 150,860 bp, exhibiting a typical quadripartite structural organization, consisting of a large single copy (LSC) region of 82,147 bp, two inverted repeat (IR) regions of 25,672 bp and a small single copy (SSC) region of 17,369 bp. The chloroplast genome contains 133 complete genes, which includes 88 protein-coding genes (88 PCGs), 8 ribosomal RNA genes (8 rRNAs), and 37 tRNA genes (37 tRNAs). Most genes occur in a single copy, while 16 genes occur in double, including all rRNAs (4.5S, 5S, 16S, and 23S rRNA), 7 tRNAs (*trnA-UGC*, *trnI-CAU*, *trnI-GAU*, *trnL-CAA*, *trnN-GUU*, *trnR-ACG*, and *trnV-GAC*), and 6 PCGs (*rps7*, *ndhB*, *ycf2*, *ycf15*, *rpl2* and *rpl23*). The overall GC content of chloroplast DNA is 37.6%, and the values of the LSC, SSC, and IR regions are 36.0%, 31.6%, and 43.0% respectively.

In order to further clarify the phylogenetic position of *D. heterophyllum*, chloroplast genomes of 11 representative Labiatae species were obtained from NCBI to reconstruct the plastome phylogeny, with *Nepeta cataria* being the outgroup. All the sequences were aligned using MAFFT v.7.313 (Katoh and Standley 2013) and maximum likelihood phylogenetic analyses were conducted using RAxML v.8.2.11 (Stamatakis 2014) under GTRCAT model with 1000 bootstrap replicates. The phylogenetic tree shows that the species of Labiatae were divided into subclades (Figure 1), one consists of the species of *Dracicephalum*, the other comprises of the species of *Scutellaria* and *Dracocephalum heterophyllum* was sister to a clade consisting of *D. moldavica* and *D. palmatum.*

**Figure 1.**
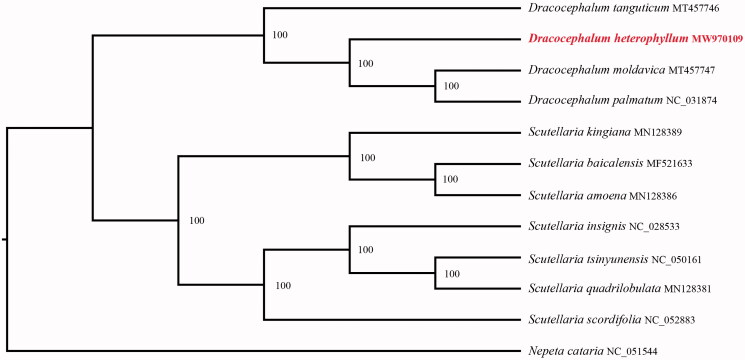
Phylogenetic relationships of *Dracocephalum* species using whole chloroplast genome. GenBank accession numbers: *Dracocephalum heterophyllum* (MW970109), *Dracocephalum moldavica* (MT457747), *Dracocephalum palmatum* (NC_031874), *Dracocephalum tanguticum* (MT457746), *Nepeta cataria* (NC_051544), *Scutellaria amoena* (MN128386), *Scutellaria baicalensis* (MF521633), *Scutellaria insignis* (NC_028533), *Scutellaria kingiana* (MN128389), *Scutellaria quadrilobulata* (MN128381), *Scutellaria scordifolia* (NC_052883), *Scutellaria tsinyunensis* (NC_050161).

## Data Availability

The data that support the findings of this study are openly available in GenBank of NCBI at https://www.ncbi.nlm.nih.gov, number MW970109. The associated BioProject, SRA, and Bio-Sample numbers are PRJNA672277, SRA: SRS8756898, and SAMN18837311, respectively.
